# The relationship between immune-related adverse events during ipilimumab monotherapy and survival outcomes among melanoma patients: A systematic review

**DOI:** 10.1177/10781552241243042

**Published:** 2024-04-05

**Authors:** Jason Sheng, Manav Nayeni, Monali Malvankar

**Affiliations:** 1 Department of Epidemiology and Biostatistics, 6221 Western University, London, ON, Canada; 2 472547 College of Osteopathic Medicine, Kansas City University, Kansas City, MO, USA

**Keywords:** Ipilimumab, melanoma, immune-related adverse events, survival

## Abstract

**Background:**

Ipilimumab disinhibits immune system activity which results in the elimination of malignant cells. An unintended consequence of ipilimumab therapy is off-target immune-related adverse events (irAEs). It has therefore been proposed that the incidence of irAEs is a manifestation of treatment effectiveness. The objective of this systematic review is to examine the relationship between irAEs and survivability among melanoma patients administered ipilimumab monotherapy.

**Methods:**

A comprehensive search was conducted across several databases which yielded a total of 2381 studies. Clinical trials and prospective studies administering ipilimumab monotherapy to melanoma patients were included. Furthermore, there was no restriction placed on publication date. After screening, five studies were included for data extraction. The primary outcome of median overall survival (OS) and the secondary outcome of OS hazard ratio were extracted from the included studies.

**Results:**

Based on qualitative analysis of the included studies, there seemed to be an association between the occurrence of non-lethal irAEs and improved survival outcomes among melanoma patients administered ipilimumab monotherapy. With that being said, the poorer survivability among patients who experienced high-grade irAEs may be the result of subsequent treatment discontinuation. Potential confounders such as corticosteroid use should be accounted for. Finally, landmark analyses may be conducted to account for immortal time bias.

**Conclusions:**

The findings from this systematic review provide evidence suggesting that the incidence of irAEs is a marker of an improved anti-tumor response.

## Introduction

Checkpoint inhibitor (CPI) therapy is a recent innovation in cancer treatment that enables the immune system to detect and eliminate malignant cells by preventing ligand binding of the immune checkpoint receptors (ICRs). Among healthy individuals, the ICR prevents an overactive immune response from attacking self-cells by downregulating immune system activity.^1^ In contrast, these receptors are upregulated among cancer patients which enables malignant cells to evade immune system detection and proliferate in the body.^2–4^ The class of anti-cytotoxic T-lymphocyte antigen-4 (anti-CTLA-4) drugs target the immune checkpoint receptor called cytotoxic T-lymphocyte antigen-4. The US Food and Drug Administration (FDA) approved ipilimumab, the first drug of this class, as a first or second-line therapy for advanced melanoma in 2011.^1,5,6^ In 2022, tremelimumab, another anti-CTLA-4 drug, was approved as a combination therapy with durvalumab for the treatment of unresectable hepatocellular carcinoma.^7^ A consequence of disinhibited immune system activity is off-target immune-related adverse events (irAEs), 0.3% to 1.3% of which are associated with fatality.^8^ Current guidelines recommend that CPI therapy be continued among patients suffering from grade 1 irAEs except for some neurologic, hematologic, and cardiac toxicities.^9^

Considering the fact that disinhibited immune system activity is associated with off-target irAEs, it would seem logical to assume that the incidence of irAEs is a marker of an improved antitumor response. Indeed, results from a previous systematic review suggest that the occurrence of mild, but not severe, irAEs is associated with improved clinical benefit.^9^ This implies that a balance must be struck between facilitating an antitumor response and managing potentially fatal irAEs. Nevertheless, the validity of these findings is limited by the retrospective nature and heterogeneity of the included studies.

Since the FDA approval of checkpoint inhibitors, several large clinical trials have been published.^10,11^ The prospective nature of these studies provides stronger evidence to determine the causality between the occurrence of irAEs and patient survival. Nonetheless, there has been no research to date that has systematically reviewed prospective studies that reported survival outcomes of patients who suffered from irAEs while undergoing anti-CTLA-4 therapy. The findings from this review will therefore inform future clinical practice regarding the management of irAEs. The objective of this systematic review is to compare survival outcomes of melanoma patients who suffered from grade 3 or 4 irAEs to those who suffered from lower grade irAEs or none at all in clinical trials and prospective cohort studies.

## Materials and methods

### Literature search strategy

Databases including MEDLINE (OVID), EMBASE (OVID), CINAHL (EBSCO), Cochrane Library (Wiley), and Web of Science (Clarivate Analytics) were searched on 1 February 2023. The search strategy from a previous systematic review^9^ was adapted to the objective of the current systematic review and optimized following consultation with a medical librarian. The search strategy can be found in Table 1. This systematic review adhered to the Preferred Items for Systematic Reviews and Meta-Analyses (PRISMA) guidelines.^12^

**Table 1.** Search strategy.

MEDLINE Search 1		
#	Search	Results
1	(CTLA-4 Antigen/ OR CTLA-4 Antigen*.tw,kf OR CTLA-4 inhibitor*.tw,kf OR CTLA4 inhibitor*.tw,kf OR cytotoxic T-lymphocyte antigen 4 inhibitor*.tw,kf OR CTLA-4 blocker*.tw,kf OR CTLA4-blocker*.tw,kf OR cytotoxic-T-lymphocyte-antigen-4- blocker*.tw,kf OR Anti-Cytotoxic T-lymphocyte antigen 4.tw,kf OR anti-CLTA4.tw,kf OR antictla-4.tw,kf OR Ipilimumab/ OR ipilimumab.tw,kf OR yervoy.tw,kf OR strentarga.tw,kf OR bms-734016.tw,kf OR bms734016.tw,kf OR mdx-010.tw,kf OR mdx010.tw,kf OR mdx-101.tw,kf OR mdx101.tw,kf OR MDX-CTLA-4.tw,kf OR tremelimumab.tw,kf)	11933
2	(drug-related side effect* OR adverse reaction* OR adverse event* OR idiosyncratic drug reaction* or immune-related OR imAR* OR imAE* OR immunotoxicity OR autoimmune toxicity).tw,kf	270516
3	(randomized controlled trial* OR randomized control trial* OR randomised controlled trial* OR randomised control trial* OR randomized clinical trial* OR randomised clinical trial* OR random control trial* OR random controlled trial* OR RCT OR clinical trial* OR phase).tw,kf OR Clinical Trial/	2219668
4	(overall survival OR progression-free survival OR progression free survival)	247861
5	1 AND 2 AND 3 AND 4	426
6	Limit 5 to humans	359
7	Limit publication type to clinical trials	304
MEDLINE Search 2		
1	(CTLA-4 Antigen/ OR CTLA-4 Antigen*.tw,kf OR CTLA-4 inhibitor*.tw,kf OR CTLA4 inhibitor*.tw,kf OR cytotoxic T-lymphocyte antigen 4 inhibitor*.tw,kf OR CTLA-4 blocker*.tw,kf OR CTLA4-blocker*.tw,kf OR cytotoxic-T-lymphocyte-antigen-4- blocker*.tw,kf OR Anti-Cytotoxic T-lymphocyte antigen 4.tw,kf OR anti-CLTA4.tw,kf OR antictla-4.tw,kf OR Ipilimumab/ OR ipilimumab.tw,kf OR yervoy.tw,kf OR strentarga.tw,kf OR bms-734016.tw,kf OR bms734016.tw,kf OR mdx-010.tw,kf OR mdx010.tw,kf OR mdx-101.tw,kf OR mdx101.tw,kf OR MDX-CTLA-4.tw,kf OR tremelimumab.tw,kf)	11933
2	(drug-related side effect* OR adverse reaction* OR adverse event* OR idiosyncratic drug reaction* or immune-related OR imAR* OR imAE* OR immunotoxicity OR autoimmune toxicity).tw,kf	270516
3	(randomized controlled trial* OR randomized control trial* OR randomised controlled trial* OR randomised control trial* OR randomized clinical trial* OR randomised clinical trial* OR random control trial* OR random controlled trial* OR RCT OR clinical trial* OR phase).tw,kf OR Clinical Trial/	2219668
4	(overall survival OR progression-free survival OR progression free survival)	247861
5	1 AND 2 AND 3 AND 4	426
6	Limit 5 to humans	359
7	Limit publication type to randomized control trials	31
EMBASE		
1	(CTLA-4 Antigen/ OR CTLA-4 Antigen*.tw,kf OR CTLA-4 inhibitor*.tw,kf OR CTLA4 inhibitor*.tw,kf OR cytotoxic T-lymphocyte antigen 4 inhibitor*.tw,kf OR CTLA-4 blocker*.tw,kf OR CTLA4-blocker*.tw,kf OR cytotoxic-T-lymphocyte-antigen-4- blocker*.tw,kf OR Anti-Cytotoxic T-lymphocyte antigen 4.tw,kf OR anti-CLTA4.tw,kf OR antictla-4.tw,kf OR Ipilimumab/ OR ipilimumab.tw,kf OR tremelimumab.tw,kf)	47769
2	(drug-related side effect* OR adverse reaction* OR adverse event* OR idiosyncratic drug reaction* or immune-related OR imAR* OR imAE* OR immunotoxicity OR autoimmune toxicity).tw,kf	468366
3	(randomized controlled trial* OR randomized control trial* OR randomised controlled trial* OR randomised control trial* OR randomized clinical trial* OR randomised clinical trial* OR random control trial* OR random controlled trial* OR RCT OR clinical trial* OR phase).tw,kf. OR Clinical Trial/	3148134
4	(overall survival OR progression-free survival OR progression free survival)	563067
5	1 AND 2 AND 3 AND 4	1497
6	Limit 5 to humans	1477
Cochrane Library		
1	(CTLA-4 Antigen (MeSH) OR CTLA-4 Antigen* OR CTLA-4 inhibitor* OR CTLA4 inhibitor* OR cytotoxic T-lymphocyte antigen 4 inhibitor* OR CTLA-4 blocker* OR CTLA4 blocker* OR cytotoxic-T-lymphocyte-antigen-4 blocker* OR Anti-Cytotoxic T-lymphocyte antigen 4 OR anti-CLTA4 OR antictla-4 OR Ipilimumab (MeSH) OR ipilimumab OR tremelimumab)	
2	(drug-related side effect* OR adverse reaction* OR adverse event OR idiosyncratic drug reaction* or immune-related OR imAR* OR imAE* OR immunotoxicity OR autoimmune toxicity)	
3	(randomized controlled trial* OR randomized control trial* OR randomised controlled trial* OR randomised control trial* OR randomized clinical trial* OR randomised clinical trial* OR random control trial* OR random controlled trial* OR RCT OR clinical trial* OR phase) OR Clinical Trial (MeSH)	
4	(overall survival OR progression-free survival OR progression free survival)	
5	1 AND 2 AND 3 AND 4	4
CINAHL		
1	(MH “CTLA-4 Antigen” OR CTLA-4 Antigen*.tw,kf OR CTLA-4 inhibitor*.tw,kf OR CTLA4 inhibitor*.tw,kf OR cytotoxic T-lymphocyte antigen 4 inhibitor*.tw,kf OR CTLA-4 blocker*.tw,kf OR CTLA4-blocker*.tw,kf OR cytotoxic-T-lymphocyte-antigen-4- blocker*.tw,kf OR Anti-Cytotoxic T-lymphocyte antigen 4.tw,kf OR anti-CLTA4.tw,kf OR antictla-4.tw,kf OR MH “Ipilimumab” OR ipilimumab.tw,kf OR tremelimumab.tw,kf)	
2	(drug-related side effect* OR adverse reaction* OR adverse event OR idiosyncratic drug reaction* or immune-related OR imAR* OR imAE* OR immunotoxicity OR autoimmune toxicity).tw,kf	
3	(randomized controlled trial* OR randomized control trial* OR randomised controlled trial* OR randomised control trial* OR randomized clinical trial* OR randomised clinical trial* OR random control trial* OR random controlled trial* OR RCT OR clinical trial* OR phase).tw,kf. OR MH “Clinical Trial”	
4	(overall survival OR progression-free survival OR progression free survival).tw,kf	
5	1 AND 2 AND 3 AND 4	0
Web of Science		
1	(CTLA-4 Antigen (MeSH) OR “CTLA-4 Antigen*” OR “CTLA-4 inhibitor*” OR “CTLA4 inhibitor*” OR “cytotoxic T-lymphocyte antigen 4 inhibitor*” OR “CTLA-4 blocker*” OR CTLA4-blocker* OR cytotoxic-T-lymphocyte-antigen-4-blocker* OR “Anti-Cytotoxic T-lymphocyte antigen 4” OR anti-CLTA4 OR antictla-4 OR Ipilimumab (MeSH) OR ipilimumab OR tremelimumab)	
2	(“drug-related side effect*” OR “adverse reaction*” OR “adverse event*” OR “idiosyncratic drug reaction*” OR imAR* OR imAE* OR immunotoxicity OR “autoimmune toxicity”)	
3	(“randomized controlled trial*” OR “randomized control trial*” OR “randomised controlled trial*” OR “randomised control trial*” OR “randomized clinical trial*” OR “randomised clinical trial*” OR “random control trial*” OR “random controlled trial*” OR RCT OR “Clinical Trial” (MeSH) OR “clinical trial*” OR phase)	
4	(“overall survival” OR “progression-free survival” OR “progression free survival”)	
5	1 AND 2 AND 3 AND 4	565

### Study selection criteria

Clinical trials and prospective cohort studies were included. There was no constraint placed on the date of publication for included articles. Studies that enrolled patients with any type of cancer who were at least 16 years old at randomization were included. The exposure group was cancer patients who suffered from grade 3 or 4 irAEs while undergoing anti-CTLA-4 therapy. The comparison group was cancer patients who suffered from grade 1 or 2 irAEs or none at all while undergoing anti-CTLA-4 therapy. The primary outcome is median overall survival (OS). The secondary outcome is the OS hazards ratio (HR).

### Data collection and analysis

#### Administration

Relevant citations were manually imported to Covidence systematic review software (Covidence, Veritas Health Innovation, Melbourne, Australia). The data extraction form was created in Microsoft Excel (Microsoft Corp.) and all information of included studies were included in this form.

#### Inclusion/exclusion procedure

**Figure 1.**
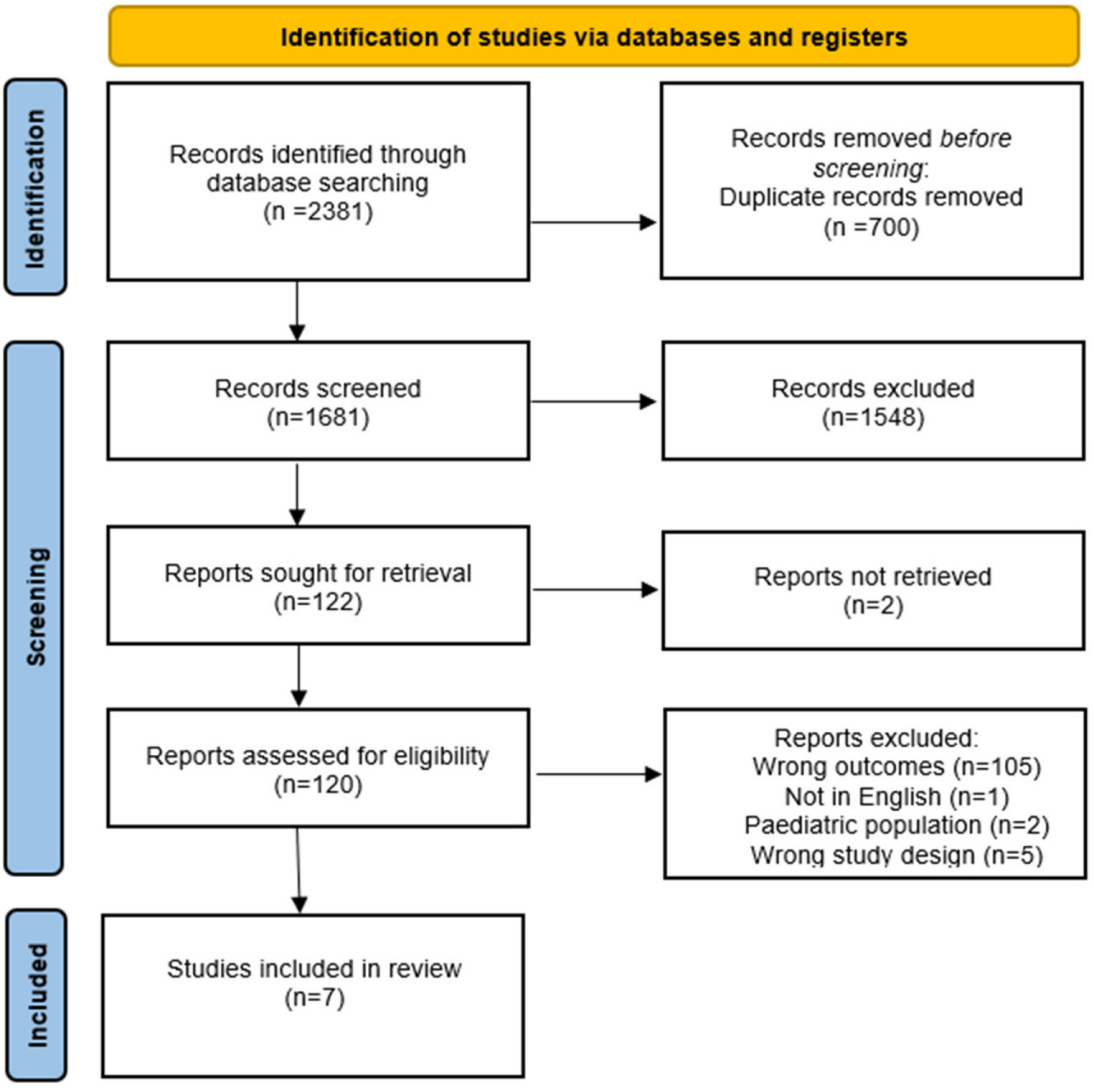
Results of database searches and final inclusion and exclusion.

MEDLINE: Medical Literature Analysis and Retrieval System Online; CINAHL: Cumulative Index to Nursing and Allied Health Literature.

Duplicates were automatically removed after uploading the studies into Covidence. In addition, a manual duplicate check was performed by a single reviewer. Level 1 screening (title screening) included any literature mentioning anti-CTLA-4 monotherapy in the title. Systematic reviews, meta-analyses, retrospective studies, case series, and case reports were excluded. Level 2 screening (abstract screening) included any literature where participants were administered anti-CTLA-4 monotherapy in the context of a clinical trial or prospective cohort study as identified through the abstract. Retrospective studies were also excluded at level 2 screening when it was not apparent based solely on a study's title and abstract whether or not a retrospective study design was implemented. Level 3 screening (full-text screening) included literature that reported median OS or OS HR among patients that suffered from any grade irAEs, grade 3 or 4 irAEs, or none at all. During each level of screening, two reviewers independently assessed the literature and conflicts were resolved. Any conflicts that remained following the discussion were referred to a third screener, after which a consensus was achieved. Cohen's kappa coefficient (*k*) was computed at each level of screening. The PRISMA flowchart displays the number of studies included and excluded at each level of screening (Figure 1).

#### Data extraction

Data were extracted by two independent reviewers. Study characteristics and demographic data of included studies were extracted including study location, median follow-up time, disease and disease stage, median age, and sex. Data regarding anti-CTLA-4 therapy administered as well as the dosage and duration were also extracted. Outcome data including median OS and OS HR as well as the respective *p*-values were extracted.

#### Assessment of risk of bias

The quality assessment of included clinical trials, after full-text screening, was conducted by two independent reviewers using the Revised Cochrane Risk-of-Bias Tool for Randomized Trials^13^ (RoB 2). These articles were assessed on five domains of potential bias including risk of bias arising from the randomization process, risk of bias due to deviations from the intended interventions, missing outcome data, risk of bias in measurement of the outcome, and risk of bias in selection of the reported result. Each domain was scored using one of the following options: low, some concern, or high. Finally, an overall risk of bias judgment was provided using one of these options. Risk of bias in prospective cohort studies was assessed using the Tool to Assess Risk of Bias in Cohort Studies by the CLARITY Group at McMaster University.^14^ This risk of bias tool asks eight questions regarding the potential risk of bias that cohort studies are particularly susceptible to and asks respondents to answer each question using one of the following options: definitely yes, probably yes, probably no, and definitely no.

## Results

### Search findings and selected studies

The search strategy yielded 2381 studies. Of these studies, 335 were identified from MEDLINE, 1477 from EMBASE, four from Cochrane, zero from CINAHL, and 565 from Web of Science. After 700 duplicates were automatically removed, 1681 studies underwent screening. After two independent reviewers conducted the title and abstract screening, a total of 1548 studies were deemed irrelevant and the remaining 121 studies proceeded to full-text screening. Based on the inclusion and exclusion criteria, five studies were included for data extraction. One of these included articles was a conference abstract that cited the results from two clinical trials. Unfortunately, only the conference abstract reported the outcome of interest. Therefore, outcome data was extracted from the conference abstract, and study characteristics and demographic data (Table 2) as well as information regarding the treatment and comparison group (Table 3) were extracted from the two associated journal articles. After the inclusion of these two journal articles, seven total studies were included for qualitative analysis. Cohen's kappa (*k*) coefficient was 0.51 and 0.14 for title and abstract screening and full-text screening respectively.

**Table 2.** Study characteristics and demographic data of included studies.

First author	Year	Study design	Clinical trial number	*N*	Study location	Follow-up time (months)	Disease (%)/stage (%)	Age (years)	Male, *n* (%)
Aamdal	2021	Clinical trial	NCT02068196	151	Norway	Median (range): 68 (0.6–76)	Melanoma (100)/M1c (64)	Median (range): 63 (27–84)	96 (64)
Hodi	2014	Clinical trial	NCT01134614	245	USA	Median (range): 13.3 (0.03–19.9)	Ipilimumab: Melanoma (100)/M1c (49.2), Ipilimumab + sargramostim: Melanoma (100)/M1c (49.6)	Ipilimumab: 64 (21–89), Ipilimumab + sargramostim: 61 (25–86)	Ipilimumab: 78 (63.9), Ipilimumab + sargramostim: 85 (69.1)
Kobayashi	2020	Cohort	N/A	174	Japan	NSCLC: 57 ± 43, Melanoma: 49 ± 46	NSCLC 62/ – (–)	NSCLC: 67 ± 10, Melanoma: 69 ± 12	NSCLC: 79 (73), Melanoma: 39 (59)
Lutzky	2009	Analysis of 2 clinical trials	CA184-008	155	Multinational	Median (Range): 10.1 (0.23–56.8)	Melanoma (100)/M1c (55.5)	Median (range): 59.0 (26.0–85.0)	80 (52)
			CA184-022	217	Multinational	10 mg/kg: median (range): 10.7 (0.43–54.5), 3 mg/kg: median (range): 8.7 (0.39–56.0), 0.3 mg/kg: median (range): 8.3 (0.53–58.7)	10 mg/kg: Melanoma (100)/M1c (51), 3 mg/kg: Melanoma (100)/M1c (50), 0.3 mg/kg: Melanoma (100)/M1c (62)	10 mg/kg: median (range): 56 (19–83), 3 mg/kg: median (range): 59 (29–78), 0.3 mg/kg: median (range): 59 (25–85)	10 mg/kg: 44 (61), 3 mg/kg: 48 (67), 0.3 mg/kg: 52 (71)
Sarnaik	2011	Clinical trial	NCT00084656	75	USA	Median (range): 30 (14–68)	Melanoma (100)/IV (61)	Median (range): 56 (21–78)	44 (59)

N/A: not applicable; NSCLC: non-small cell lung cancer; IV: intravenous.

**Table 3.** Treatment and comparison group.

First author	Year	Study design	Clinical trial number	Treatment	N	Dosage, duration	Comparison	n	Dosage, duration
Aamdal	2021	Clinical trial	NCT02068196	Ipilimumab	151	3 mg/kg every 3 weeks for 4 total doses	N/A	N/A	N/A
Hodi	2014	Clinical trial	NCT01134614	Ipilimumab + placebo	122	10 mg/kg every 3 weeks for 4 total doses followed by maintenance therapy every 3 months	Ipilimumab + sargramostim	123	Ipilimumab 10 mg/kg on day 1 + sargramostim, 250 μg, on days 1 to 14 of a 21-day cycle
Kobayashi	2020	Cohort	N/A	Ipilimumab	24	3 mg/kg every 3 weeks for 4 total doses	N/A	N/A	N/A
Lutzky	2009	Analysis of 2 clinical trials	CA184-008	Ipilimumab	155	10 mg/kg every 3 weeks for 4 total doses followed by maintenance therapy every 3 months	N/A	N/A	N/A
			CA184-022	Ipilimumab	10 mg/kg: 73, 3 mg/kg: 72, 0.3 mg/kg: 72	10 mg/kg, 3 mg/kg, or 0.3 mg/kg every 3 weeks for 4 total doses followed by maintenance therapy every 3 months	N/A	N/A	N/A
Sarnaik	2011	Clinical trial	NCT00084656	Ipilimumab	10 mg/kg: 50, 3 mg/kg: 25	3 mg/kg or 10 mg/kg every 6–8 weeks for 52 weeks followed by maintenance therapy every 3 months for those eligible	Ipilimumab + tyrosinase, gp100, and MART-1	50	Ipilimumab 3 or 10 mg/kg every 6–8 weeks for 52 weeks followed by maintenance therapy every 3 months for those eligible + 1 mg each of tyrosinase, gp100, and MART-1 every 2 weeks for 6 doses, every 4 weeks for the next 4 doses, and 12 weeks between the final 2 doses

### Study characteristics

Data extraction was performed for all included studies. Study characteristics and demographic data can be found in Table 2. Five studies were clinical trials, one was a conference abstract and another was a prospective cohort study. A total of 1017 patients were recruited across all six primary studies. The studies took place in Norway, USA, and Japan. Ipilimumab monotherapy was administered to melanoma patients across all studies at similar dosages and durations (Table 3). Four studies reported median OS (Table 4) and one study reported OS HR (Table 5). Furthermore, Kobayashi et al.^15^ were only able to collect median OS data for six patients with pituitary irAEs and six patients without pituitary irAEs.

**Table 4.** Median OS.

First author	Year	Study design	Clinical trial number	Subgroup	Disease (%)/stage (%)	n	Median OS, months (95%CI)	*p*-value
Hodi	2014	Clinical trial	NCT02068196	Ipilimumab + sargramostim: complete intent to treat population	Melanoma (100)/M1c (49.6)	123	17.5 (14.9–NR)	One-sided: 0.01
				Ipilimumab: complete intent to treat population	Melanoma (100)/M1c (49.2)	122	12.7 (10.0–NR)	
				Ipilimumab + sargramostim: censor 15 lethal adverse events	Melanoma (100)/– (–)	123	NR (17.5–NR)	One-sided = 0.008
				Ipilimumab: censor 17 lethal adverse events	Melanoma (100)/– (–)	122	19.6 (12.6–NR)	
				Ipilimumab + sargramostim: censor 2 lethal adverse events with treatment-relation of “possibly,” “probably,” and “definitely”	Melanoma (100)/– (–)	121	NR (14.9–NR)	One-sided = 0.03
				Ipilimumab: censor 7 lethal adverse events with treatment-relation of “possibly,” “probably,” and “definitely”	Melanoma (100)/– (–)	115	14.3 (11.1–NR)	
Kobayashi	2020	Cohort	N/A	With pituitary irAE	Melanoma (100)/– (–)	6	NC	NC
				Without pituitary irAE	Melanoma (100)/– (–)	6	5.14 (2.80–7.48)	—
Lutzky	2009	Analysis of 2 clinical trials	CA184-008 and CA184-022	Median OS from day 81 among patients with any grade irAE	Melanoma (100)/– (–)	—	14.8 (10.0–21.7)	—
				Median OS from day 81 among patients with no irAE within 12 weeks	Melanoma (100)/– (–)	—	8.21 (5.29–13.7)	—
				Median OS from day 81 among patients with grade ≥2 irAE	Melanoma (100)/– (–)	—	13.6 (5.78-NR)	—
				Median OS from day 81 among patients with no grade ≥2 irAE within 12 weeks	Melanoma (100)/– (–)	—	11.3 (7.95–15.8)	—
Sarnaik	2011	Clinical Trial	NCT00084656	Ipilimumab + tyrosinase, gp100, and MART-1	Melanoma (100)/– (–)	50	NR	—
				Ipilimumab	Melanoma (100)/– (–)	10 mg/kg: 50, 3 mg/kg: 25	NR	—
				With significant irAE	Melanoma (100)/– (–)	28	NR	—
				No irAE	Melanoma (100)/– (–)	47	58	—

NR: not reached; NC: not calculated (too few patients to calculate reliably); irAEL irAE: immune-related adverse event; OS: overall survival.

**Table 5.** OS HR.

First author	Year	Subgroup	*n*	OS HR (95% CI)
Aamdal	2021	Grade 3 or 4 irAE within 3 months after treatment initiation	20	0.50 (0.30–0.83)
		Any grade irAE	106	0.71 (0.47–1.08)

OS HR: overall survival hazard ratio; irAE: immune-related adverse event.

### Risk of bias

Three out of the five clinical trials were rated as being at high risk of bias arising from the randomization process (Table 6). These studies were single-arm clinical trials and were therefore subject to selection bias. The single prospective cohort study that was included after the full-text review did not compare baseline characteristics between patients who suffered from irAEs compared to those who did not (Table 7). As such, the results from this study are susceptible to selection bias. This study was also rated as being at risk of bias for co-intervention bias. This is because patients who suffered from irAEs were treated with corticosteroids to manage symptoms. Finally, all included studies were subject to lead time bias where improved survival time is inappropriately attributed to patients who were diagnosed earlier. These biases are discussed in greater detail in the discussion section.

**Table 6.** Risk of bias assessment of clinical trials.

	Aamdal 2021	Hodi 2014	O'Day 2010	Sarnaik 2011	Wolchok 2010
Domain 1: Risk of bias arising from the randomization process	High	Some concerns	High	High	Low
Domain 2: Risk of bias due to deviations from the intended interventions (effect of assignment to intervention)	Some concerns	Some concerns	Some concerns	Some concerns	Low
Domain 2: Risk of bias due to deviations from the intended interventions (effect of adhering to the intervention)	Some concerns	Some concerns	Some concerns	Some concerns	Low
Domain 3: Missing outcome data	Low	Low	Low	Low	Low
Domain 4: Risk of bias in measurement of the outcome	Low	Low	Low	Low	Low
Domain 5: Risk of bias in selection of the reported result	Low	Low	Low	Low	Low
Overall risk of bias	Some concerns	Low	Some concerns	Some concerns	Low

**Table 7.** Risk of bias assessment of cohort studies.

	Kobayashi 2020
Was selection of exposed and non-exposed cohorts drawn from the same population?	PY
Can we be confident in the assessment of exposure?	Y
Can we be confident that the outcome of interest was not present at the start of the study?	Y
Did the study match exposed and unexposed for all variables that are associated with the outcome of interest or did the statistical analysis adjust for these prognostic variables?	N
Can we be confident in the assessment of the presence or absence of prognostic factors?	PY
Can we be confident in the assessment of the outcome?	Y
Was the follow-up of cohorts adequate?	Y
Were co-interventions similar between groups?	N

PY: probably yes; Y: yes; N: no.

### Survivability

In the current systematic review, median OS was lowest in the cohort study conducted by Kobayashi et al.^15^ Although statistical significance was not calculated, there was an improvement in median OS among patients with any grade irAEs compared to those without irAEs in the two clinical trials analyzed by Lutzky et al.^16^ However, caution should be exercised when interpreting these findings as they are almost certainly subject to immortal time bias. For some unspecified reason, Lutzky et al.^16^ calculated the median OS from day 81 onwards. Sarnaik et al.^17^ reported an exceptional median OS of 58 months among those without irAEs. Unfortunately, there was insufficient data to calculate the median OS among those who were afflicted with significant irAEs.^17^ In the study conducted by Hodi et al.,^18^ there seemed to be a small improvement in median OS among patients who were administered ipilimumab after censoring lethal adverse events resulting in death compared to the complete intent to treat population.

## Discussion

An inadvertent consequence of increased immune system activity induced by CPI therapy is off-target irAEs, a fraction of which results in fatality. Given the relatively recent innovation of CPI therapy, there has been a paucity of scientific literature that has synthesized prospective data from clinical trials. Current guidelines primarily rely on low-quality observational data, established guidelines, case series, and case reports.^9^ Expert consensus was relied upon in instances where there was a lack of evidence.^9^ Considering this, the objective of the current systematic review is to assess the survivability of melanoma patients who experienced high-grade irAEs while undergoing ipilimumab therapy compared to those who experienced low-grade irAEs or none at all.

Current treatment guidelines recommend that CPI be temporarily withheld and corticosteroids (initial dose of 0.5 to 1 mg/kg/day of prednisone) be administered to treat most grade 2 toxicities.^9^ In addition to temporary discontinuation of CPI therapy, more intensive corticosteroid treatment (1 to 2 mg/kg/day of prednisone or 1 to 2 mg/kg/day of intravenous methylprednisolone) is recommended for patients suffering from grade 3 toxicities.^9^ Infliximab may be administered if symptoms do not improve within 48 to 72 h of steroid administration.^9^ CPI therapy may be resumed when irAEs improve to grade 1 toxicity.^9^ Grade 4 toxicities often require permanent discontinuation of CPI therapy.^9^

A previous systematic review suggested a positive association between irAEs and survival among patients undergoing CPI therapy.^19^ These findings were replicated in the current systematic review among melanoma patients administered ipilimumab monotherapy. Data from the current systematic review also suggests that treatment discontinuation following high-grade irAEs may cause poorer survival outcomes as opposed to high-grade irAEs per se. Nevertheless, lethal irAEs were associated with poorer survival outcomes. This suggests that an enhanced immune response induced by CPI therapy must be balanced against the risk of potentially fatal irAEs. Indeed, milder irAEs affecting the endocrine as opposed to the hepatic or pulmonary system were associated with improved survival.^19^

The current systematic review contributes to the existing literature by replicating previous findings using data from clinical trials and prospective cohort studies that are less susceptible to bias. Additionally, the patient populations of the included studies in the current systematic review were homogenous with regard to the type of cancer and treatment which facilitated comparison between studies. In contrast, much of the research synthesized in current guidelines did not restrict studies based on these characteristics.^9^ Findings from the current systematic review also suggest that treatment exposure is likely a confounder in the relationship between irAEs and survival outcomes which may explain conflicting results observed in the literature.

There are nonetheless limitations of the current systematic review. Most notably, only a few studies were randomized. This may have introduced selection and immortal time bias, the latter of which occurs when patients who are diagnosed earlier are inappropriately attributed with longer survival time. This is addressed in clinical trials by randomizing baseline characteristics including duration of disease across treatment arms which enables comparison between groups. Having said that, immortal time bias may be an inevitable limitation of the current systematic review since survival outcomes were compared between patients within the same treatment arm. As a result, the process of randomization was rendered redundant. Landmark analyses have been proposed to address this issue.^20^ The current systematic review, such as other similar reviews, also suffered from co-intervention bias since patients who suffered from irAEs were often treated with corticosteroids. This likely had an inconsequential impact on validity since corticosteroid administration was only associated with poorer survival outcomes among patients treated for palliative indications or brain metastases but not among patients treated for irAEs.^21–23^ This is an example of selection bias since the former group required higher doses of corticosteroids for longer durations of time (≥10 mg of prednisone equivalent for ≥10 days).^21,23^ In fact, patients with brain metastases suffer from poorer survival outcomes.^24,25^

In future studies, investigators should report baseline characteristics of patients who suffered from irAEs as well as those who did not to assess the comparability of groups and address potential selection bias. Investigators should moreover adjust for corticosteroid use. Given the nuanced relationship between severity of irAEs and survival outcomes, it would be useful to stratify survival outcomes by severity of irAEs. Lastly, landmark analyses can be used to account for potential immortal time bias.

In the current systematic review of prospective studies administering ipilimumab monotherapy to melanoma patients, there was a positive association between irAEs and survival outcomes. Future research should be conducted to investigate whether or not CPI therapy discontinuation is an intermediate variable in the relationship between high-grade irAEs and poorer survival outcomes. Nonetheless, ipilimumab is still a relatively new treatment and a definitive conclusion on the relationship between irAEs and survival can not be reached at the present moment.
